# Supplementary material for: Seminoma and Embryonal Carcinoma Footprints Identified by Analysis of Integrated Genome-Wide Epigenetic and Expression Profiles of Germ Cell Cancer Cell Lines
Source: PLoS One. 2014 Jun 2;9(6):e98330. doi: 10.1371/journal.pone.0098330 (PMC4041891; doi:10.1371/journal.pone.0098330)
Supplement: File S1 — ZIP file containing DMRforPairs output for significant regions. Please start from the html files. (ZIP) [file pone.0098330.s008.zip › figures/21925.pdf]

RegionID: 21925, chr19:38182773−38183456−M_values

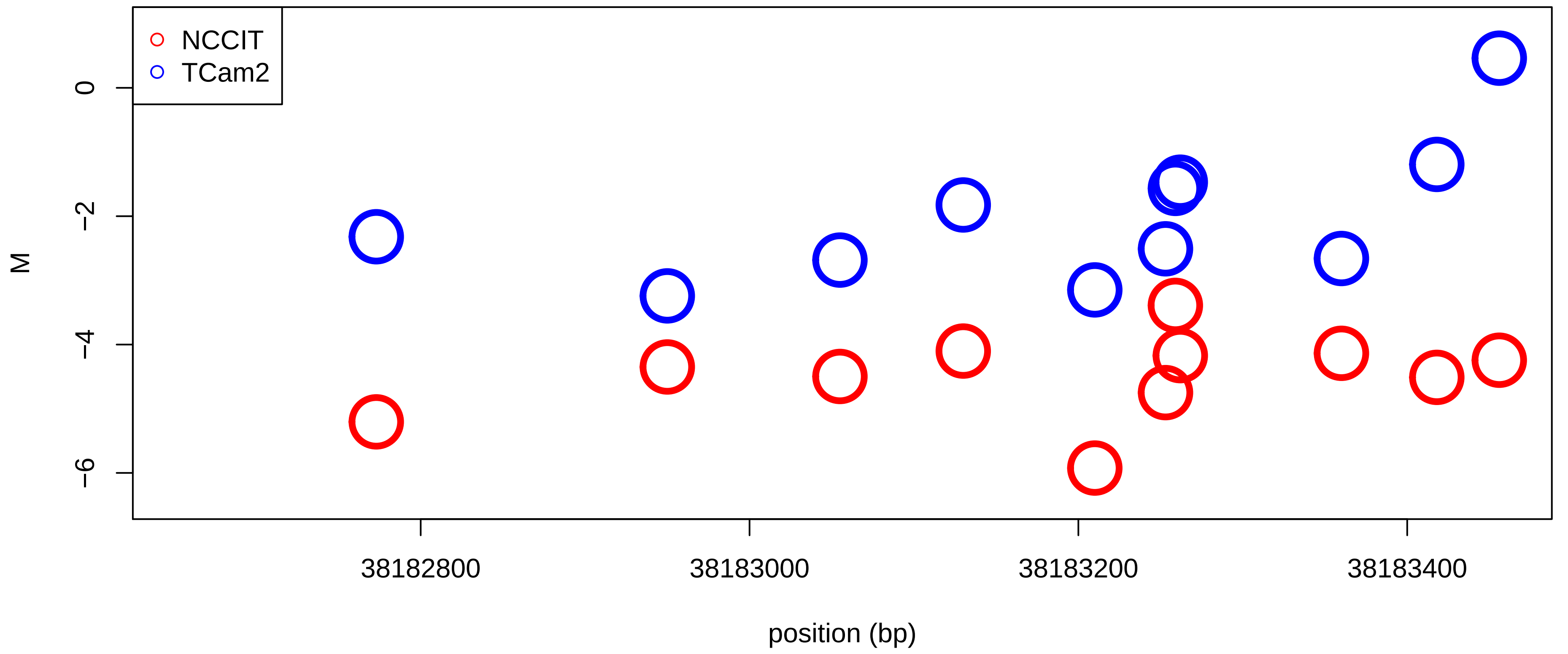

RegionID: 21925, chr19:38182773−38183456−Beta_values

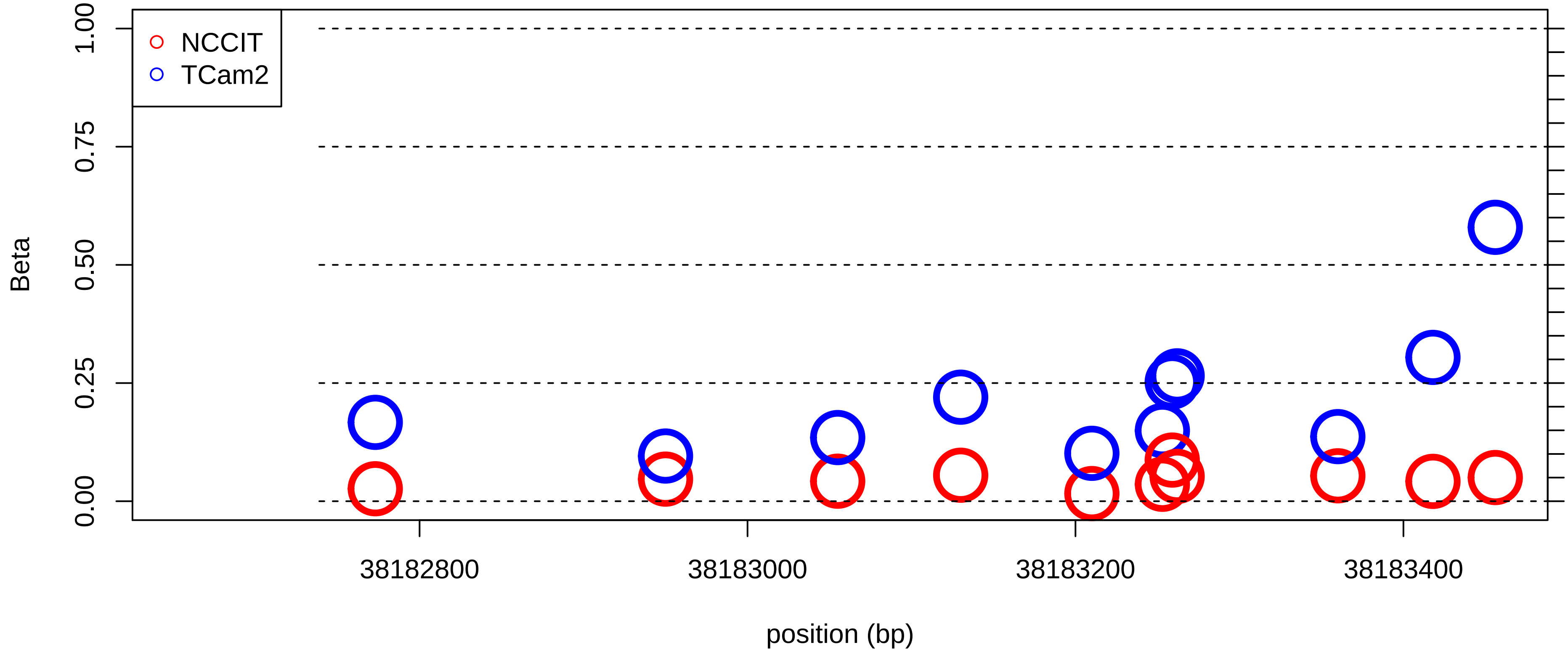

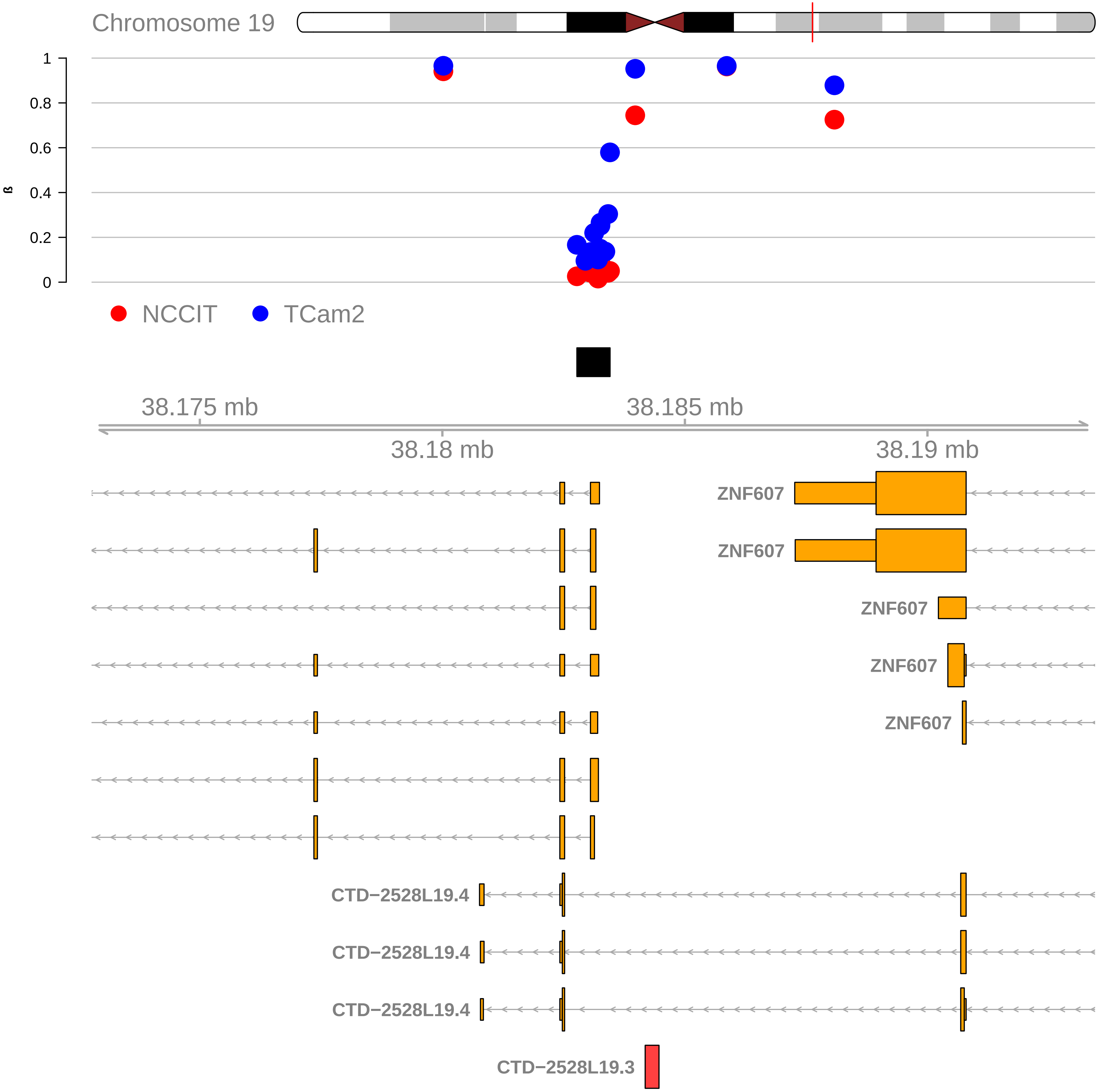

Chromosome 19
ß
1
0.8
0.6
0.4
0.2
0
NCCIT
TCam2
38.175 mb
38.185 mb
38.18 mb
38.19 mb
ZNF607
ZNF607
ZNF607
ZNF607
ZNF607
CTD−2528L19.4
CTD−2528L19.4
CTD−2528L19.4
CTD−2528L19.3
